# Assessing the number of contributors to two-, three-, and four-person mixtures in forensic casework

**DOI:** 10.3325/cmj.2011.52.654

**Published:** 2011-10

**Authors:** Theresa Caragine

**Affiliations:** Office of Chief Medical Examiner of the City of New York, The Department of Forensic Biology, New York, NY, USA

**In Reply:** The purpose of Perez et al (1) is to describe allelic patterns observed in a large set of purposeful two-, three- and four-person mixtures in order to assist in estimation of the number of contributors to mixtures encountered in evidence samples where the source(s) of the samples are unknown. This work supplements previously published articles on the determination of number of contributors in a mixture that are based on simulated data (2-4) and represents the first effort of its kind using empirical results. We welcome discussion such as that of Jamieson et al (5). We anticipate that we and others will test the guidelines presented here using additional mixtures of known origin and that the guidelines will evolve as a result of these discussions. The guidelines presented in Perez et al (1) were developed using The New York City Office of Chief Medical Examiner (NYC OCME) casework protocols, including routine duplicate amplification of high-template mixtures, triplicate amplification of low-template mixtures, and reliable quantification of the amount of template DNA in each sample (6). We are cognizant that the specifics of such guidelines will vary from laboratory to laboratory, as different equipment, protocols, and interpretation procedures are used. Recognizing the aim of this work and realizing that the guidelines are not universal eliminates many of the concerns presented in (5). Below, we address the remaining issues.

The reason to assess the number of contributors, even for a four-person mixture, is of course the need to include this parameter in the Likelihood Ratio (LR) hypothesis. Although treating a true four-person mixture as a three-person mixture may be conservative (7), tools to differentiate between three- and four- person mixtures are helpful, with the goal of making the best estimate, not simply the most conservative. The practical application of an LR approach to mixture statistics is not individualization. Rather this approach provides weight of evidence for a scenario in which a person of interest is a contributor compared to a scenario in which the person of interest is not a contributor.

Another concern was the level of detail provided regarding the test samples. In order to reflect the range of results encountered in forensic casework, over 728 samples covering a variety of DNA template amounts from a diverse set of donors mixed in different ratios were examined. The number of samples amplified with a specific mixture ratio and number of contributors, as well as whether the sample contained high-template or low-template amounts of DNA was noted in the methods section. The ethnic composition of the pool of contributors used was also specified. To list the exact amount of template DNA amplified for each mixture ratio and combination of donors and their individual ethnicity for each of the 728 samples as suggested by the authors of this letter (5) would have required a full page table. More importantly, when examining an evidence sample, the mixture ratio determination can only yield an approximation and rules should not be established to apply to just one specific ratio.

Alternatively, it is helpful to look for general characteristics in mixtures. To illustrate whether different mixture ratios significantly affect the determination of the number of contributors to a mixture, samples were divided into six separate graphs (Figures 4A-4F) of two-, three-, and four-person mixtures composed of high-template or low-template amounts of DNA. These data sets were then further subdivided into mixtures with ratios of contributors that were similar or dissimilar, and the numbers of different alleles observed, a key parameter in assessing the number of contributors to mixtures, were shown. For each of the six groupings, the number of different alleles labeled was not substantially different for mixtures with similar ratios as compared to those with more extreme ratios. Contrary to what the authors of the letter state, this and other findings may be replicated by studying similar general groupings.

The letter further critiques the experimental design using defined DNA dilutions. Although there is some uncertainty in the exact amount of DNA amplified, the DNA concentration measured using our in-house assay is a good estimate (6). To accommodate possible variation within a quantitation assay, DNA from each contributor used to create mixtures was measured in triplicate three times for a total of nine measurements. To address the stochastic effects, samples were created and amplified two or three times.

There was no subjective editing of data and allele calls in this study. All filters and analysis thresholds employed are programmed into the software and were not altered from sample to sample. Also, at the NYC OCME evidence extracts are routinely tested using multiple amplifications and thus multiple runs are available for analysis.

The authors of the letter speculate that data may have been selected to fit a preconceived hypothesis. This was not the case. Each purposeful mixture was evaluated as if it was a forensic unknown using the proposed guidelines, a strength of this study. The described characteristics of three- and four-person mixtures were never observed in the set of two- and three-person mixtures studied, respectively. Some samples, however, did not meet the specified criteria and had to be classified as two-person mixtures even though they were created with DNA from three individuals, for example. The frequency that this occurred was clearly stated.

The reality of forensic casework mixtures, especially those containing low amounts of DNA, is that allelic drop-out may occur for one or more components, which may mask the true number of contributors. In our study, we categorized these types of mixtures more conservatively (7), and then presented a second set of error assessments based on the frequency that mixtures, for example, appeared to be three-person mixtures but were made from the DNA of four, not three, individuals.

Lastly, we recognize that although the study with touched items represents a blind data set, one shortcoming is that the actual number of contributors amplified is not known since one may not recover DNA from all persons who handle an item. Another large data set of purposeful mixtures would be required. Nevertheless, we maintain that our study provides useful tools to determine the number of contributors to mixtures processed in our laboratory. Based on these studies, work by our colleagues in their own laboratories may customize these guidelines for their use and/or develop more enhanced mechanisms to address the issue.
